# Double minute chromatin bodies in a sub-ependymal glioma.

**DOI:** 10.1038/bjc.1968.82

**Published:** 1968-12

**Authors:** K. Kucheria

## Abstract

**Images:**


### DOUBLE MINUTE CHROMATIN BODIES IN A SUB-EPENDYMAL GLIOMA

KIRAN KUCHERIA

*From the Department of Morbid Anatomy, Institute of Child Health, Guilford St., London W.C.1*

Received for publication August 7, 1968

DURING chromosome studies (*in vitro*) of human solid tumours, double minute chromatin bodies were observed in a proportion of cells derived from a sub-ependymal-glioma. Although similar bodies have been described in other human solid tumours (Lubs and Salmon, 1965; Cox, Yuncken and Spriggs, 1965), they have not been reported in this particular type of neoplasm.

### *Case report*

The child presented with a short history of lethargy, irritability and vomiting. Clinical examination revealed neck stiffness, papilloedema and possibly choroidal tubercles. Skull radiogram showed separation of sutures. C.S.F. contained raised proteins and low sugar.

Further investigations showed a normal blood picture and E.S.R. The Mantoux test was negative. The C.S.F. was essentially normal. X-ray of the skull showed that the vault was thin and there was suture diastasis.

*Histological examination.*—A tumour showing a marked perivascular pattern composed of large cells with prominent processes. Mitotic figures were not seen. There was some calcification. The appearances were those of a sub-ependymal glioma.

*Chromosome studies.*—Pieces of tumour were set up for tissue culture using the method of Jensen, Gwatkins and Biggers (1964). The cells were processed for chromosome examination 5 weeks after the primary culture was set up. Only 38 metaphases were suitable for counting and analysis.

The chromosome number showed a definite mode at 46 (Table I). In addition, double minute chromatin bodies (D.B.) were observed in 11 of the cells examined (33%). From the 11 cells that were karyotyped, 5 were apparently normal but 4 of them contained one double minute chromatin body. The other 6 cells were pseudodiploid and 2 of them contained one double minute chromatin body (Fig. 1, 2). One cell with 45 chromosomes had two double minute chromatin bodies.

TABLE I.—*Chromosome Counts*

| Chromosome numbers | | | | | | | Total cells |
|---|---|---|---|---|---|---|---|
| 39 | 40 | 44 | 45 | 46 | 92 | . | — |
| 1 | 1 | 1 | 1 | 33 | 1 | . | 38 |

EXPLANATION OF PLATES

FIG. 1.—Metaphase spread showing one double minute chromatin body.
FIG. 2.—Karyotype of the pseudodiploid cell shown in Fig. 1.

1

Kucheria.

2

Kucheria.

### DISCUSSION

Lubs and Salmon (1965) made similar type of observations in a case of medulloblastoma and Cox *et al.* (1965) in 3 cases of neuroblastoma, 2 rhabdomyosarcoma and 1 bronchial carcinoma. The double minute chromatin bodies were found, however, in large numbers in the majority of the cells, and could have been formed by repeated duplication of these bodies, whereas in the present case, they were observed only one per cell. The chromosomes of D and G group in the present case show very prominent satellites which correspond with the size and morphology of these minute bodies. It is possible that in the cells where only one double minute chromatin body was found it could have been formed by the process of separation of the satellites from their respective chromosome.

Since the patient has not received any kind of therapy, it is unlikely that these double bodies could be chromosome fragment formed by breakage. None of the analysed cells had any chromosome breaks or gaps or acentric fragments.

The presence of these double minute chromatin bodies does not seem to have any direct relationship to the number of karyotypic distribution of the chromosomes since they were present in normal and abnormal karyotypes.

The author wishes to thank Dr. A. E. Claireaux for his most helpful suggestions. The work was supported by the British Empire Cancer Campaign for Research.

### REFERENCES

Cox, D., Yuncken, C. and Spriggs, A. I.—(1965) *Lancet*, ii, **55**, 58.
Jensen, F. C., Gwatkin, R. B. L. and Biggers, J. D.—(1964) *Expl Cell Res.*, **34**, 440.
Lubs, H. A. and Salmon, J. H.—(1965) *J. Neurosurg.*, **22**, 160.


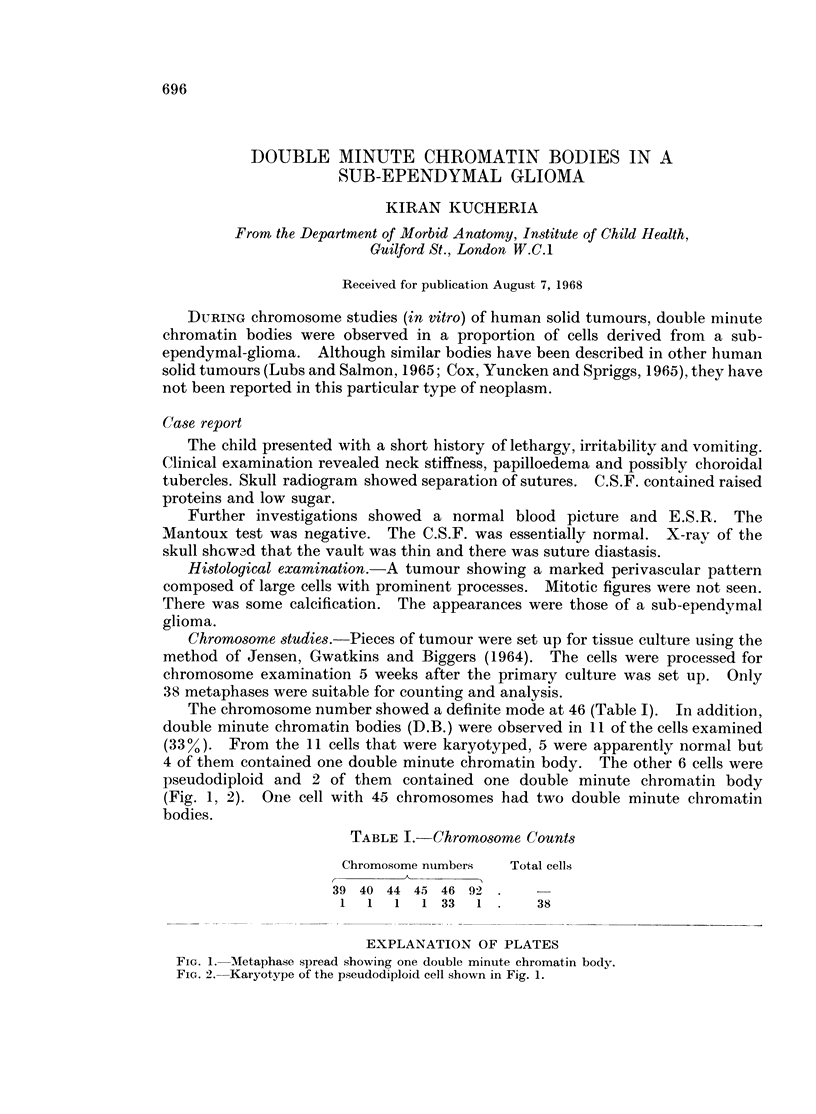

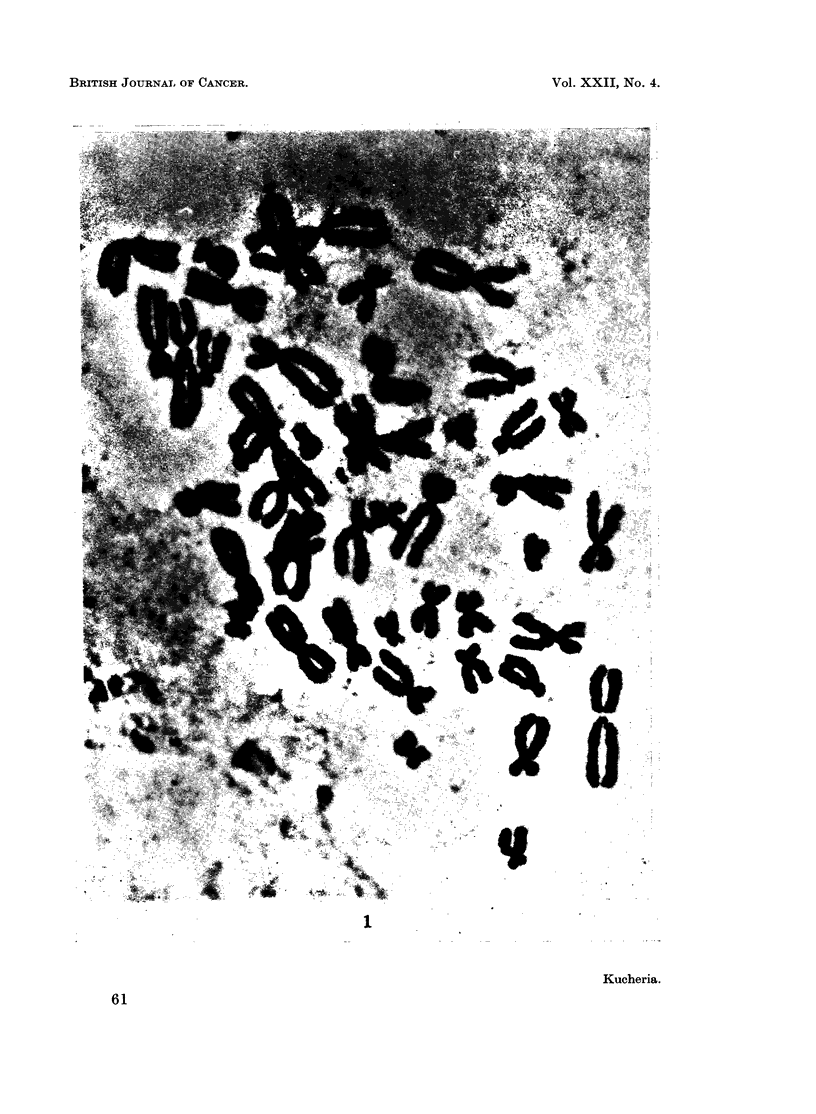

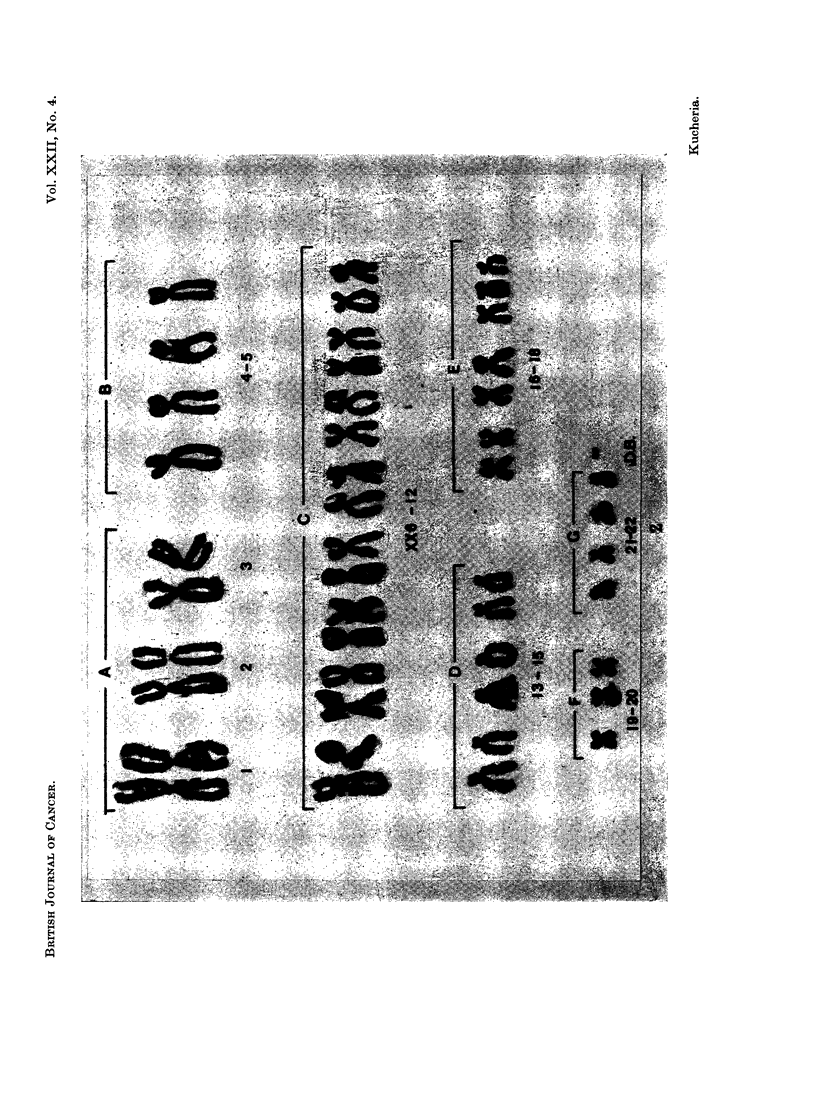

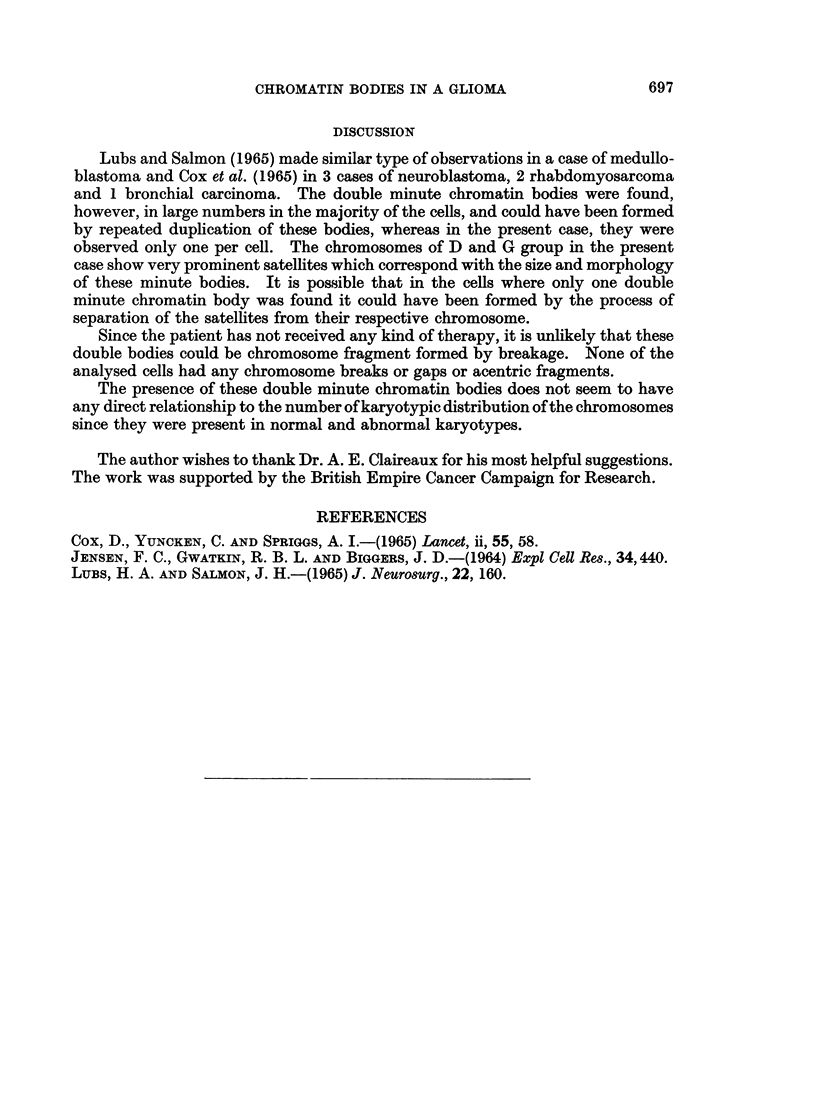


## References

[OCR_00176] COX D., YUNCKEN C., SPRIGGS A. I. (1965). MINUTE CHROMATIN BODIES IN MALIGNANT TUMOURS OF CHILDHOOD.. Lancet.

[OCR_00178] JENSEN F. C., GWATKIN R. B., BIGGERS J. D. (1964). A SIMPLE ORGAN CULTURE METHOD WHICH ALLOWS SIMULTANEOUS ISOLATION OF SPECIFIC TYPES OF CELLS.. Exp Cell Res.

[OCR_00179] LUBS H. A., SALMON J. H. (1965). THE CHROMOSOMAL COMPLEMENT OF HUMAN SOLID TUMORS. II. KARYOTYPES OF GLIAL TUMORS.. J Neurosurg.

